# Supplementary material for: Content-rich biological network constructed by mining PubMed abstracts
Source: BMC Bioinformatics. 2004 Oct 8;5:147. doi: 10.1186/1471-2105-5-147 (PMC528731; doi:10.1186/1471-2105-5-147)
Supplement: Additional File 2 — The original results of the above study (non-essential files are deleted to keep the file size under the limit set by BMC bioinformatics). [file 1471-2105-5-147-S2.bz2 › chilibotAdditionalFile2/dip20/540ID9211894E10893/html/AR_FOS.html]

 


 **AR** and **FOS** 
  
Found 62 abstracts in PubMed, retrieved 20.  
 

 What does Google say? 
 PDF only 
| .edu only 

---

**Interactive relationship** (e.g. stimulation, inhibition, etc)

**Inhibitory relationship**- Compared with MR rats, pup exposed  **AR**  rats engaged in less pup licking, and all  **AR**  rats showed significant reductions in c  **fos**  immunoreactivity in the medial preoptic area and the parietal and piriform cortices.  Ref: 12492299 Behav Neurosci, 2002
- Pre treatment with the selective beta 3  **AR**  antagonist SR59230A resulted in a significant decrease in the number of  **Fos**  positive cells in all those areas compared with rats treated with CL316243 alone.  Ref: 10963888 Neurosci Lett, 2000
**Neutral relationship**- Neurons in these brain areas are rich in androgen receptors  **AR**  and express  **FOS**  immunoreactivity in response to mating.  Ref: 12689608 Psychoneuroendocrinology, 2003
- The  **AR**  min group showed the greatest difference in  **Fos**  lir compared with the MR groups.  Ref: 12492299 Behav Neurosci, 2002

**Non-interactive relationship** (e.g. studied together, co-existance, homology, etc.)

- Since activator protein 1 AP 1, a heterodimer of c jun and c  **fos**  proteins, is known to inhibit binding of  **AR**  to ARE by protein protein interaction with  **AR** , the findings in the present study suggest a possible involvement of AP 1 in the antiandrogenic effects of PAHs acting as AhR agonists.  Ref: 12799773 Arch Toxicol, 2003
- We have conducted experiments investigating the effects of i.c.v. administration of the selective beta 3  **AR**  agonist CL316243 on  **Fos**  expression to determine whether beta 3  **AR**  stimulation affects neurones within specific brain nuclei.  Ref: 10963888 Neurosci Lett, 2000
- We report that, identically to 17beta estradiol or dihydrotestosterone, but differently from raloxifene, estren alters the activity of Elk 1, CCAAT enhancer binding protein beta C EBPbeta, and cyclic adenosine monophosphate response element binding protein CREB, or c Jun c  **Fos**  by an extranuclear action of the ER or  **AR** , resulting in activation of the Src Shc ERK pathway or downregulation of JNK, respectively.  Ref: 12782668 J Clin Invest, 2003
- The alpha 2  **AR**  agonists, clonidine and UK14304 also had no effect on basal IEG levels, while blockade of alpha 2 ARs by methoxyidazoxan significantly increased NGFI A and c  **fos**  expression, but decreased c jun mRNA levels.  Ref: 11072104 Brain Res Mol Brain Res, 2000
- After experiencing social stress the brain area within the medial preoptic area was removed for analysis of c  **fos**  and androgen receptor  [ **AR** ]  mRNA expression.  Ref: 12131378 J Urol, 2002
- Although the AP 1 c Jun c  **Fos**  system and NcoR have been reported to interact with and repress the activity of some steroid receptors, c Jun, c  **Fos** , c Jun c  **Fos** , nor NcoR function was consistently affected by the absence or presence of residues 628 646, implying that the  **AR**  hinge region exerts its silencing effects in a manner independent of these corepressors.  Ref: 11102454 J Biol Chem, 2001
- Effect of prenatal androgen receptor  [ **AR** ]  antagonist or aromatase inhibitor on sexual behavior, partner preference and neuronal  **Fos**  responses to estrous female odors in the rat accessory olfactory system.Many socially relevant odors are detected in rodent species by the vomeronasal organ and subsequently processed by the accessory olfactory system.  Ref: 11897260 Physiol Behav, 2002
- ... inhibition of c  **Fos** , c Jun, HB EGF,  **AR** , and VEGF mRNA by this compound was incomplete 41 65%.  Ref: 12016209 J Biol Chem, 2002
